# Effect of Porosity on Mechanical Properties of 3D Printed Polymers: Experiments and Micromechanical Modeling Based on X-ray Computed Tomography Analysis

**DOI:** 10.3390/polym11071154

**Published:** 2019-07-05

**Authors:** Xue Wang, Liping Zhao, Jerry Ying Hsi Fuh, Heow Pueh Lee

**Affiliations:** 1 Department of Mechanical Engineering, National University of Singapore, Singapore 117575, Singapore; 2 National Metrology Centre, 1 Science Park Drive, Singapore 118221, Singapore

**Keywords:** 3D printing, X-ray computed tomography, mechanical properties, micromechanical modeling

## Abstract

Additive manufacturing (commonly known as 3D printing) is defined as a family of technologies that deposit and consolidate materials to create a 3D object as opposed to subtractive manufacturing methodologies. Fused deposition modeling (FDM), one of the most popular additive manufacturing techniques, has demonstrated extensive applications in various industries such as medical prosthetics, automotive, and aeronautics. As a thermal process, FDM may introduce internal voids and pores into the fabricated thermoplastics, giving rise to potential reduction on the mechanical properties. This paper aims to investigate the effects of the microscopic pores on the mechanical properties of material fabricated by the FDM process via experiments and micromechanical modeling. More specifically, the three-dimensional microscopic details of the internal pores, such as size, shape, density, and spatial location were quantitatively characterized by X-ray computed tomography (XCT) and, subsequently, experiments were conducted to characterize the mechanical properties of the material. Based on the microscopic details of the pores characterized by XCT, a micromechanical model was proposed to predict the mechanical properties of the material as a function of the porosity (ratio of total volume of the pores over total volume of the material). The prediction results of the mechanical properties were found to be in agreement with the experimental data as well as the existing works. The proposed micromechanical model allows the future designers to predict the elastic properties of the 3D printed material based on the porosity from XCT results. This provides a possibility of saving the experimental cost on destructive testing.

## 1. Introduction

Additive manufacturing is labeled as one of the breakthrough innovations since the 19^th^ century according to the 2015 World Intellectual Property Report, which regards its impact on the manufacturing industry as the same as the airplane impact on the transportation industry [1]. It has been popularly identified as a revolutionary technology reshaping the manufacturing world, having the advantages such as low cost of raw material, fast prototyping for customized small batches, and high flexibility in designing complex structures [2,3]. As the most widely used additive manufacturing technology [3], fused deposition modeling (FDM) offers an efficient technique of fabricating thermoplastics, which makes it highly popular for modeling, prototyping, and production applications [4,5,6,7].

In the FDM process, the thermoplastic filament as feedstock is melted through a liquefier head, extruded via a computer controlled nozzle, and subsequently deposited and solidified on the platform to build the part in a layer-by-layer manner. The fabrication process itself is a thermal one introducing heterogeneities in micro/meso length scale, especially voids and pores [8,9], of which the size, shape, and spatial distribution are highly dependent on the process parameters. Such voids and pores may affect the internal structure of the deposited materials and, in turn, affect the mechanical properties of the final product.

There are extensive experimental and numerical studies in literature correlating the void or pore details in mesostructures and the mechanical properties of the products fabricated by FDM process. For example, References [3,8] conducted experiments to study how the presence of voids in the mesostructures affects the mechanical properties of the printed material. References [10,11] proposed multiscale finite element models using representative volume elements (RVE) to investigate the relationship between mechanical properties and process parameters by taking into account the mesostructures containing voids. However, very few studies have investigated the microscopic internal pores which may also significantly affect the mechanical properties of FDM processed parts.

One technique for characterizing the microscopic details of the internal pores is X-ray computed tomography (XCT), currently recognized as the most effective nondestructive test method for measuring the internal features of the products fabricated by additive manufacturing [12]. As a volumetric measurement tool, XCT has demonstrated its capability in evaluating the three dimensional microscopic details of pores, such as shape, density, and distribution, in 3D printed metallic materials such as stainless steel [13] and titanium alloy [14], as well as analyzing the porosity in metallic powder feedstock [15]. Nevertheless, few studies have been found on XCT characterizations for the three dimensional internal features of FDM processed polymers where the microscale pore analysis is important for understanding the mechanical properties of the product.

This paper presents systematic studies to quantify the correlation between the microscopic details of internal pores and the macroscopic mechanical properties of 3D printed polymeric material from experimental aspects and micromechanical modeling. As a fundamental step, three dimensional details of microscale pores in polymeric materials fabricated by the FDM process, including size, density, shape, and spatial location of the pores, were characterized quantitatively using X-ray computed tomography. Subsequently, mechanical tests were conducted to characterize the material properties which were then correlated with the porosity (defined as density of the pores) as well as the process parameters. Most importantly, based on the actual details of the internal pores characterized by XCT, a micromechanical finite element model was developed for FDM users to predict the elastic properties of the product. It is worthy to note that classical micromechanical models based on continuum mechanics, such as the generalized self-consistent method [16] and the Mori–Tanaka method [17], do not attempt to capture the microscopic details of the internal feature of materials.

The results of the present study aim to provide future designers a methodology for predicting the elastic properties of FDM processed materials using the porosity results from XCT. This may save the material from undergoing destructive testing.

## 2. Experimental Methodology

### 2.1. Specimens Preparation

In this study, a desktop FDM 3D printer (MOMENT, Moment Co. Ltd., Geumcheon-gu, Seoul) was adopted for fabricating the mechanical test specimens using a polylactic acid (PLA) filament supplied by Meka 3D Printing Pte Ltd, Singapore. The geometry of the specimens was designed to follow tensile specimen Type IV in ASTM D638 testing guidelines (ASTM D638-14, Standard Test Method for Tensile Properties of Plastic) with the specimen thickness chosen to be 4 mm. The 3D model of the specimens were created in a CAD package (SolidWorks, Dassault Systèmes SolidWorks Corp., Waltham, MA, USA), exported as a stereolithography (STL) file and subsequently loaded into 3D printer slicing software (Simplify3D, Simplify3D Inc., LLC, Blue Ash, OH, USA) to generate the G-code which the 3D printer used to print out each test specimen.

As reported in the literature [18,19], the FDM process is directly affected by the process parameters such as raster orientations, extrusion width, extrusion temperature [20], infill pattern [21], and printer-head speed [22,23], which lead to variations of the internal structure as well as mechanical properties of the printed materials. A comprehensive review for the influence of the process parameters on the final products has been given in [19].

**Figure 1:**
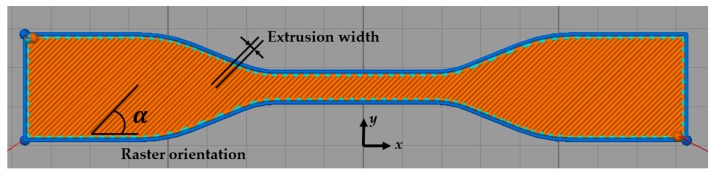
An illustration of the raster angle and extrusion width from Simplify3D toolpaths. The building direction is along the *z*-axis.

Among these process parameters, raster orientations (the direction of the raster pattern relative to the loading of the part, usually in either unidirectional or crisscross patterns) and extrusion width (also known as raster/bead width, defined as the width of the raster pattern) are extensively recognized as two of the main aspects affecting the porosity and mechanical behavior of the 3D printed products [19,21]. Thus in this study, we had selected raster orientation (in terms of raster angle with respect to the *x*-axis) and extrusion width as the parameters to vary in the FDM process. The parameter values of interest were categorized into five sets given in Table 1, where three specimens were printed for each set of process parameters to study the variations of the FDM process. An illustration of raster angle and extrusion width from Simplify3D toolpaths is shown in Figure 1. The layer thickness was fixed as the commonly used value 0.2 mm, the infill density was fixed as 100% for all the sets to ensure a dense structure giving rise to sufficient material strength and a retraction vertical lift for the extrusion nozzle was set as 0.6 mm to avoid the nozzle scratching the previously deposited layer during movement. The other process parameters remain untouched as the default values: Nozzle temperature 67 °C, platform temperature 210 °C, infill pattern rectilinear, printing bed speed 40 mm/s, to name a few.

### 2.2. Quantitative XCT Characterization for Internal Pores

**Figure 2:**
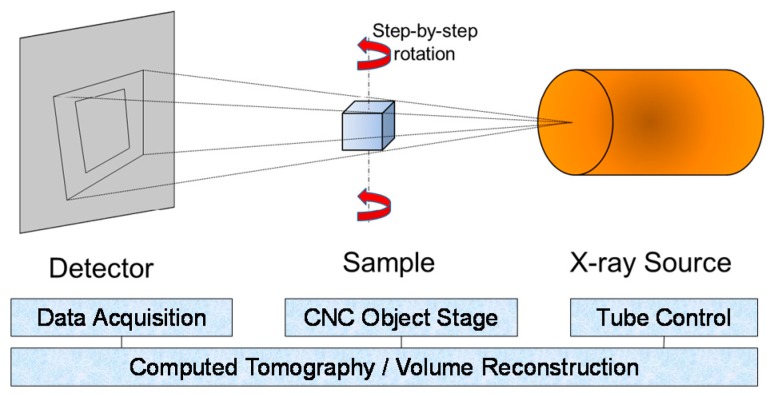
Schematic illustration of a cone-beam X-ray computed tomography (XCT) system.

**Figure 3:**
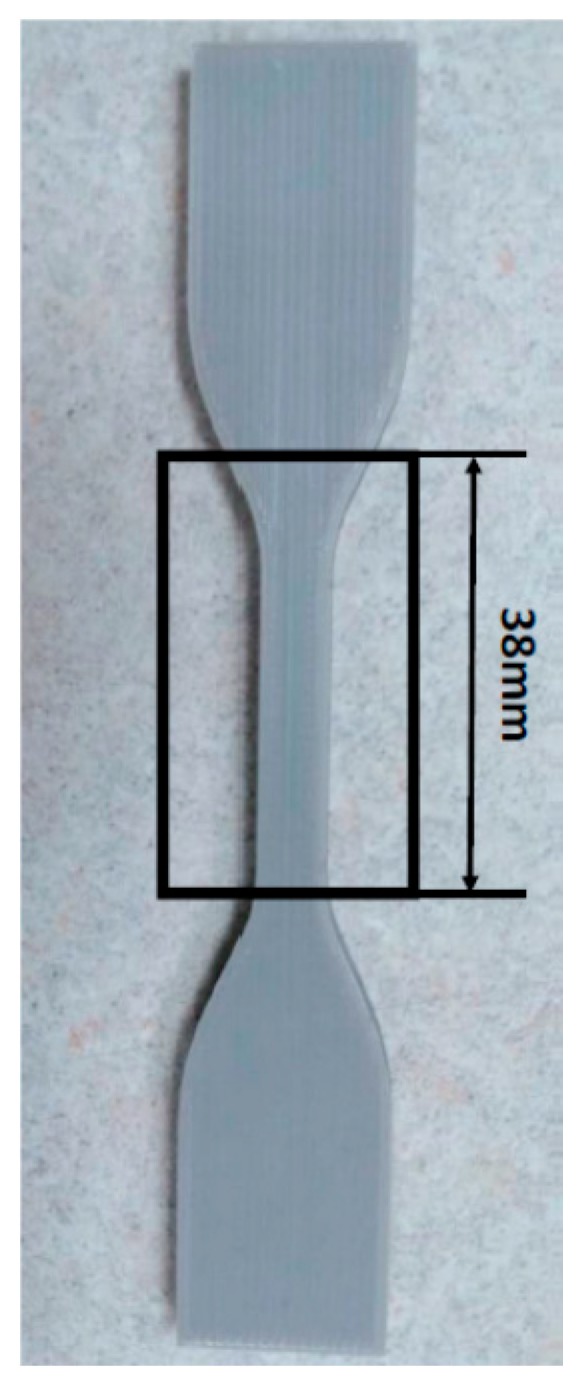
An illustration of the scanned area for XCT analysis in the PLA specimens.

XCT is considered as the most promising non-destructive method to quantitatively reveal the 3D features and internal defects [13,24,25,26] of a complex part. It utilizes a chromatic X-ray cone beam as a non-contact radiation media to disclose the 3D geometrical features in a nondestructive approach. As shown in Figure 2, an X-ray beam penetrates through the sample, and attenuates due to scattering and absorption caused by physical geometry and the material intrinsic properties. The areal array detector captures the signal of the transmitted X-ray beam, and therefore records the 2D attenuation distribution image corresponding to the scanned angle. Through a 360° rotation, a significant number of 2D images, normally several thousand images, are acquired and thus a high resolution 3D volume can be reconstructed by a computing algorithm. The resolution was dependent on the scanning settings, as well as the relative positions of part, X-ray, and the detector. In this study, GE Nanotom M was used to characterize the internal features of the 3D printed PLA material. The XCT system was first calibrated with ruby ball bars to ensure traceable scales and high precision measurements. The voltage and current was applied at 120 kV and 100 µA, respectively. The AM parts were located close to the rotational center of the precision actuator to minimize the decenter-caused measurement errors; meanwhile, they were placed at the closest possible position to the X-ray source to maximize the imaging magnification and resolution. The area under investigation for a representative PLA specimen is illustrated in Figure 3.

After scanning and reconstruction, VG studio Max3.0 (Volume Graphics GmbH, Heidelberg, Germany) was used for surface determination and volume analysis. The Porosity Analysis module was applied to investigate voxel data sets for internal imperfections such as pores and inclusions and to provide detailed analysis results with information on each individual defect as well as overall statistical information.

The pore analysis procedure consists of two steps: (a) Each voxel is checked as to whether it might be part of a pore/void or not, and groups of connected defect candidates are created, and (b) each group of defect candidates is checked as to whether it matches the parameters specified, mainly covering pore/defect size range and probability. The VGDefX algorithm [27] was used for this study.

### 2.3. Mechanical Testing

**Figure 4:**
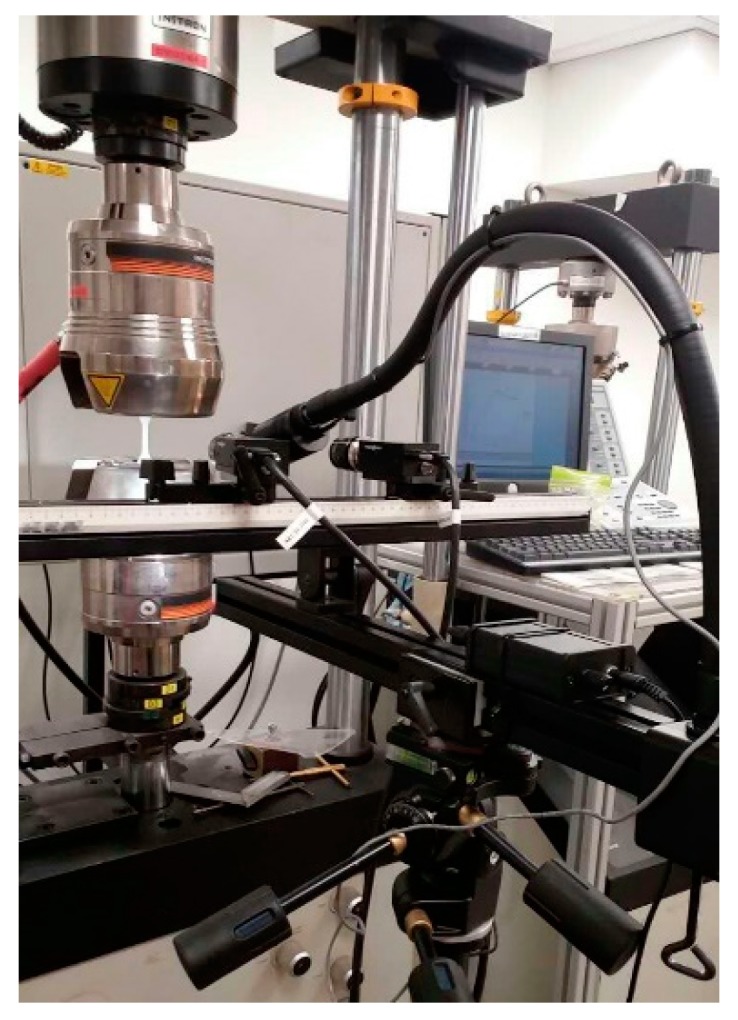
Experimental setup for the tensile tests.

The three specimens for each set of process parameters given in Table 1 were tested by following ASTM D638 testing guidelines. A universal testing machine (Instron 5900 series, Instron Corp., Norwood, MA, USA) with a load cell 100KN as well as 3D digital image correlation (DIC), a full-field deformation measurement technique, was used to test the mechanical properties of the specimens. To utilize the DIC technique, high contrast speckle patterns in black and white were applied to the specimens before the tensile tests. Each specimen was then clamped at the two ends in the universal testing machine by two pairs of hydraulically controlled jaws, which were separated at a speed of 2 mm/min until the specimen fractured. During the whole test process, the specimen images were captured at a rate of 10 Hz by high resolution DIC cameras, and simultaneously, the load data used for calculating the stress (that is, tensile load divided by the cross-sectional area of the specimen) were recorded by the testing machine at the same rate. After the tensile tests, the DIC images were analyzed using a DIC data processing system (VIC-3D, Correlated solutions, Inc., Irmo, SC, USA) to calculate the averaged strains in a rectangular area in the center of each tensile specimen. Such a methodology provides the strain values in both longitudinal and transverse directions in good precision [28]. Subsequently, the stress–strain curves were obtained and the mechanical properties were processed using a script (MATLAB, The MathWorks, Natick, MA, USA). The experimental setup for the tensile tests is given in Figure 4.

### 2.4. Statistical Analysis

Statistical analysis was conducted to evaluate the consistency of the mechanical properties of the test specimens fabricated by the MOMENT 3D printer. As introduced in the previous sections, three specimens were printed for each set of process parameters given in Table 1 and individually tested for characterizing the mechanical properties. For each set of process parameters, the mean, standard deviation, and coefficient of variations for the mechanical properties of the three specimens were calculated to analyze the fluctuation of the statistical data.

## 3. Results and Discussions

### 3.1. Results for XCT Characterizations

The three-dimensional details of the internal pores in the PLA specimens, including porosity, size, shape, and spatial location of the pores, were characterized using the XCT technique. The results are presented below.

#### 3.1.1. Pore Size Distribution

To investigate the size distribution of internal pores for the specimens, predefined upper and lower pore size limits for the XCT detection were set before the characterizations. The lower pore size limit was determined by the scanning resolution which was selected as 30 μm (three voxels). A larger value of upper pore size limit allows larger pores into the calculation of porosity, but leads to a more costly computation. To make a compromise between the accuracy of the porosity calculation and the computation time, three various upper pore size limits (abbreviated as “UPL” below) were used for analyzing the pore size distribution as well as calculating the porosity of the specimens. In this study, the pore size was characterized by the diameter of the circumscribed sphere of the pore.

The pore size distributions analyzed using the various upper pore size limits are presented in Table 2 for a selected specimen in Set E. Note that the smallest pore size detected was always around 0.0388 mm which was determined by the scanning resolution. The pore size was separated into seven intervals based on the statistical measurement by the XCT equipment and the number of pores in each interval was counted and is presented in Table 2. It is obvious for all the three values of UPL that a large majority of pores had very small sizes below 0.2 mm, which contributed over 99% of the total number of pores. The medium pore sizes between 0.2 mm and 0.6 mm occupied only around 0.75% of the pore population and the pores with sizes larger than 0.8 mm occupied less than 0.1%. This may imply that the PLA material fabricated by the FDM process tends to generate a major population of smaller pores.

**Figure 5:**
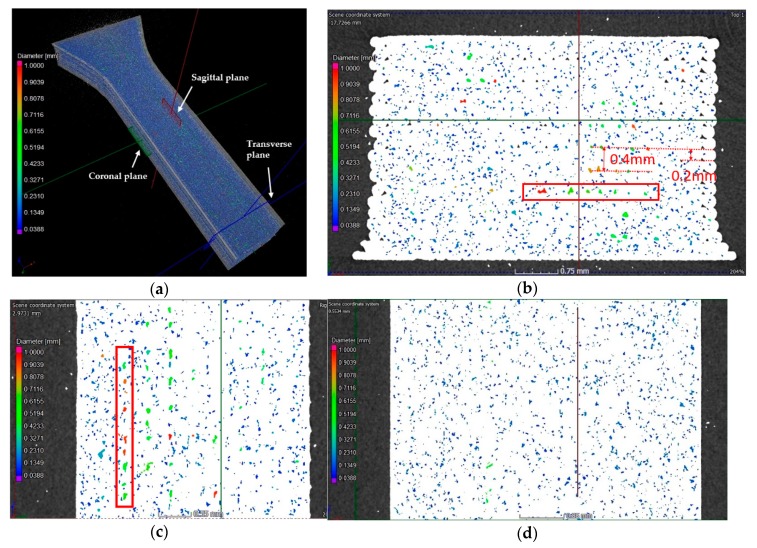
Pore distribution for UPL = 1 mm characterized by X-ray computed tomography (XCT) of three-dimensional visualization in (**a**) and two-dimensional visualization in (**b**) the transverse plane, (**c**) the sagittal plane, and (**d**) the coronal plane. The red slim rectangular in (**b**) and (**c**) mark the selected pores of large size between two adjacent layers of the specimen. The distance between the l inearly aligne d large pores in (**b**) and (**c**) was measured as 0.4 mm, which is twice of the layer thickness of 0.2 mm.

**Figure 6:**
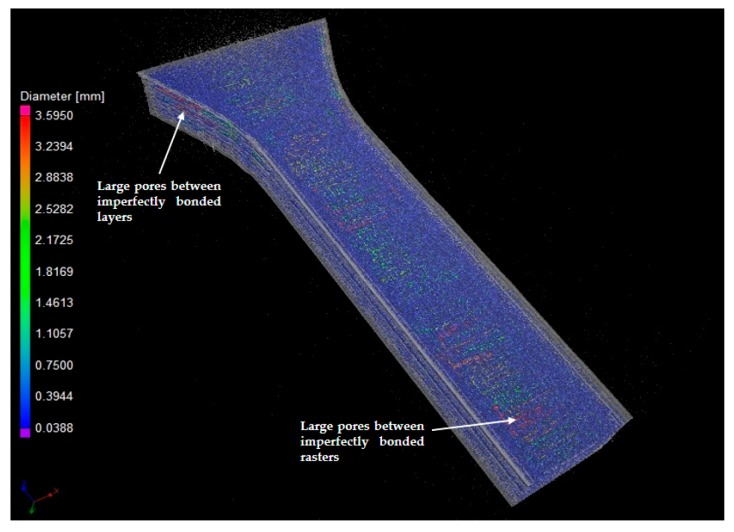
Three-dimensional visualization of pore distribution for UPL = 3.6 mm. Representative large pores between the imperfectly bonded layers and rasters are highlighted.

It was also clear that the pore size distribution for the three UPLs was quite similar, although the analysis with the limits of 1.8 mm and 3.6 mm captured a very small amount of large pores which were not quantified by the limit of 1 mm. The spatial locations and size distributions of the pores for UPL = 1 and 3.6 mm, respectively, are shown in the three-dimensional XCT images in Figure 5a and Figure 6. It was visualized that the pores were densely distributed over almost the whole PLA specimen—this was not surprising considering that the thermoplastic filament was highly affected by the heating and cooling iterations during the printing process. Clearly the porosity analysis with UPL = 3.6 mm captured a number of larger pores presenting between the bonded layers and rasters, which were probably induced by the imperfect bonding but were not captured by the analysis with smaller UPL.

Using the pore size distributions in Table 2, the values of porosity were correspondingly calculated, as presented in Table 3. As expected, the analysis using the three UPLs resulted in very close values of porosities. This indicates that the upper pore size limit 1 mm may be sufficient for quantifying the pores and it was therefore used for characterizing the porosity and pore size distributions of the rest of the specimens.

To better visualize the pore distribution, the three-dimensional XCT image in Figure 5a was mapped to transverse, sagittal, and coronal planes, respectively, as illustrated in Figure 5b–d. The two-dimensional images showed clearly that the large population of pores with small sizes below 0.2 mm had irregular shapes. It is noteworthy that the large pores tended to have more regular shapes (ellipsoid-like or cuboid-like) and tended to appear in a linear array between two pairs of the 0°/90° crisscross layers, as illustrated in Figure 5b. This denotes that the large pores may have been caused by the imperfect bonding between adjacent pairs of the crisscross layers.

#### 3.1.2. Correlation between FDM Process Parameters and Specimen Porosity

The porosity results for the selected specimens are given in Table 4 for various raster orientations and in Table 5 for various extrusion widths. All results were calculated with the upper pore size limit predefined as 1 mm.

Here we correlated the porosity with the raster orientations first. For the unidirectional raster orientations, the specimens printed in 0° were observed to have smaller porosity compared to those printed in 90°. For the crisscross raster orientations, the specimens in 45°/−45° obviously have smaller porosity than the ones in 0°/90° orientations. Also, the porosity values for all the specimens in 0°/90° (given in Table 5) were larger compared to the specimens in the other three orientations (see Table 4). That is, the specimens in 0°/90° had the largest density of pores among all of the raster orientations in both unidirectional and crisscross patterns. When it came to the specimens printed in different extrusion widths, the results showed that the specimen with the larger extrusion width had a lower porosity when compared.

The observations above may be better understood by investigating the pore size distributions of each set of specimens. The range of the pore size for each specimen was divided into five categories and the pore size distribution was compared by counting the number of pores in each category. Note that all the specimens here had the same range of pore size, that is, from 30 μm (the lower pore size limit determined by the selected resolution) to 1 mm which was the selected upper pore size limit. The representative comparison results are given in Table 6, where the data for sets A, B, and C came from the specimen #1 in each set. It was observed that there were less pores in each of the pore size categories if the specimen had a smaller porosity. This may provide a direct explanation for the porosity results by taking into account that specimens in 0°/90° raster orientations have more pores in each pore size category compared to the specimens in the other three raster orientations; also the specimens with a 0.24 mm extrusion width had more pores within each pore size category than the ones with a 0.48 mm extrusion width. Another important observation is that the specimens in all of the sets had similar size distributions, that is, over 99% of the pore sizes were located between 0 and 0.2 mm. This provided an important reference for the micromechanical modeling in Section 3.3.

**Figure 7:**
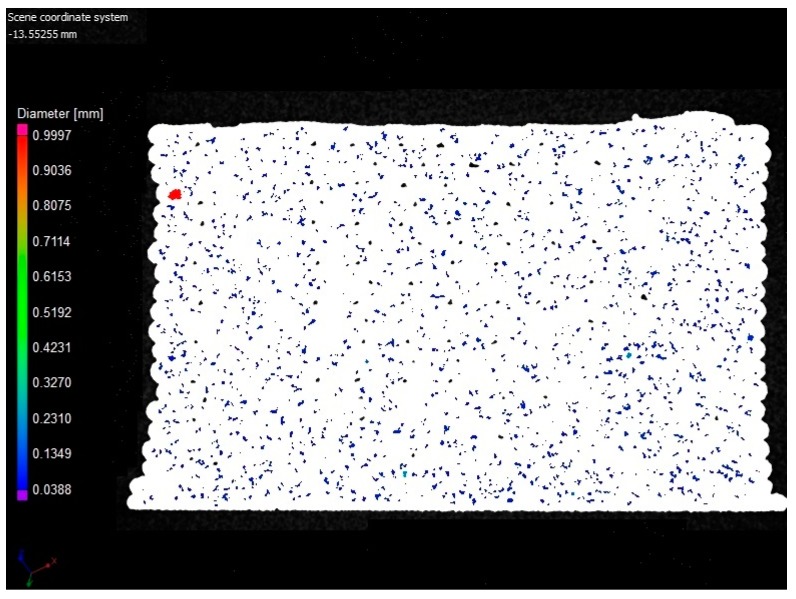
A representative two-dimensional visualization for the specimens in the 0° raster orientation in the transverse plane.

The pore shapes and spatial locations were also investigated from XCT results for the specimens in all of the five sets. First of all, the specimens in 0°, 90° and 45°/−45° were observed to have very similar patterns of pore shapes and spatial distributions. Taking a specimen with 0° raster orientation as an example, a representative XCT image for visualizing the pore distribution for the specimen is given in Figure 7. Another interesting finding is that most of the large pores (having ellipsoid-like shapes) in the specimens printed in the raster orientations 0°, 90° and 45°/−45°, which have relatively small porosity compared to the specimen in 0°/90°, tended to appear at the bonding areas between the specimen outline and infill rasters, instead of the regions between adjacent bonded layers in 0°/90° orientations as in Figure 5. Moreover, the large pores between the specimen outline and infill rasters in 0°, 90° and 45°/−45° orientations appear to be fewer than those large pores between the adjacent layers in 0°/90° orientations. This is further analyzed together with the results of mechanical properties in the next section.

### 3.2. Results for Mechanical Testing

**Figure 8:**
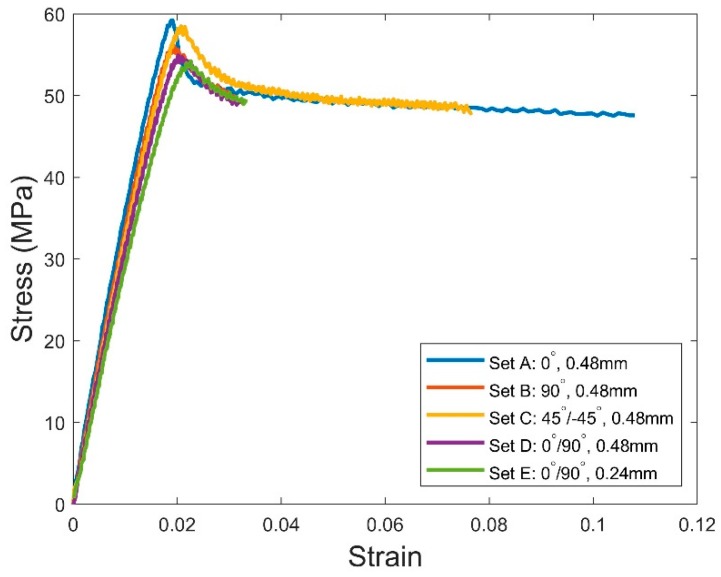
Stress-strain curves for representative specimens in each set of process parameters.

From the statistical analysis, the mechanical properties characterized by the tensile tests are presented in Table 7 in terms of the mean and standard deviation of the three specimens in each set of process parameters. Correspondingly, the stress–strain curves for the representative specimen in each set are given in Figure 8. At first glance, the 3D printed PLA material exhibited significant anisotropic behaviors in the tensile properties. By selecting the different raster orientations in the printing, the percentage differences of the Young’s modulus, ultimate tensile strength, and the strain at fracture for the specimens respectively reached 17.5%, 10.8%, and 70.4%. The highest anisotropy existed in the mechanical properties between set A and set B, as well as set C and set D. More specifically, specimens printed in 0° and 45°/−45° raster orientations had obviously higher elastic modulus, ultimate strength, and ductility compared to those in 90° and 0°/90° orientations. The anisotropy is not surprising if we look back on the porosities presented in Table 4 and Table 5—the PLA materials printed in 90° and 0°/90° orientations had larger porosities (as well as more pores) compared to the ones in 0° and 45°/−45°, and therefore gave rise to stronger stress concentrations which led to weaker mechanical properties. Similarly, as expected, the tensile tests showed that the specimens printed with a 0.48 mm extrusion width (having lower porosity and less pores) exhibited better mechanical properties compared to those printed with a 0.24 mm extrusion width.

Another important observation is that all specimens printed in 0°/90° raster orientations had worse mechanical properties compared to those in 0°, 90°, and 45°/−45° raster orientations. This can be explained by considering the spatial locations of the large pores in the specimens of different raster orientations. As observed in the last section, the large pores for 0°/90° specimens tended to appear between the bonded areas of adjacent layers; however, the pores of large sizes for 0°, 90°, and 45°/−45° specimens were found between the specimen outlines and infill rasters. It suggests that 0°/90° specimens tend to have worse bonding quality, giving rise to lower mechanical properties, than the other raster orientations.

**Figure 9:**
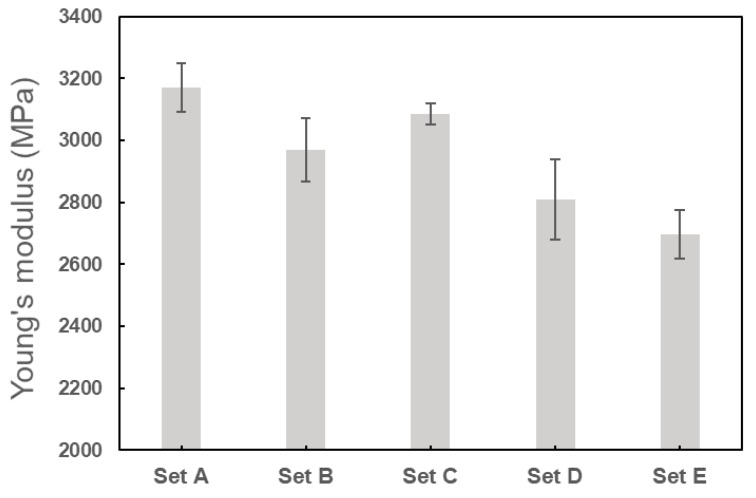
Experimental data of Young’s modulus for each set of specimens.

**Figure 10:**
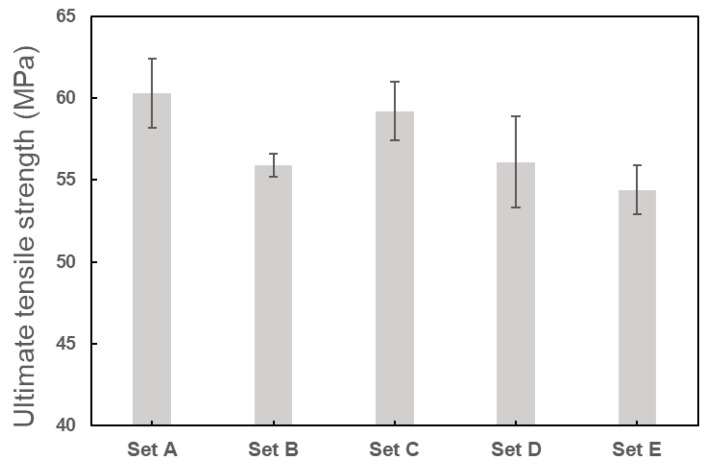
Experimental data of ultimate tensile strength for each set of specimens.

The variations for Young’s modulus, Poisson’s ratio, and ultimate tensile strength for the three specimens in each set were quantified using standard deviations in Table 7. The Poisson’s ratio was observed to vary very little for the three specimens in each parameter set. Also, the mean value of Poisson’s ratio in each parameter set was approximately equal to 0.33, which was almost independent of the process parameters. The coefficient of variations of Young’s modulus and ultimate tensile strength was, respectively, up to 4.6% and 5.0%, both of which were considered reasonably accepted. The variations of Young’s modulus and ultimate tensile strength are illustrated in Figure 9 and Figure 10, respectively.

### 3.3. Micromechanical Modelling Based on Quantitative XCT Analysis

A micromechanical finite element model is proposed in this section to predict the elastic properties of 3D printed PLA material based on the actual microscopic details of the pores characterized by XCT. The model was developed through an two dimensional analysis of a periodic representative volume element (RVE), a micromechanical technique widely used in the literature for estimating the macroscopic behavior of composites which consist of a matrix containing microscale inhomogeneities, such as pores or fibers [10,29,30]. The identification of such a composite material into an RVE is not unique. One popular identification method is to assume that the inhomogeneities are uniformly distributed in the matrix and have the same geometry [31,32]. This method is easy to implement but clearly not adequate for quantifying the size distribution and the spatial location of pores in FDM processed materials.

**Figure 11:**
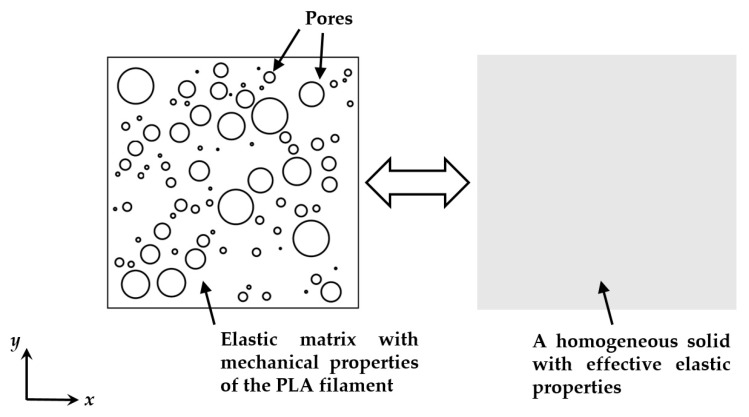
A sketch of the representative volume element and the equivalent homogeneous solid.

Here we propose an identification methodology based on the internal structures of the 3D printed PLA material, as illustrated in Figure 11, where the RVE was modeled as a square elastic matrix containing *N* pores whose sizes were determined according to the actual pore sizes characterized by XCT. The pores were assumed to have circular shapes since the actual pore sizes were quantified in the XCT analysis by measuring the diameter for the circumscribed sphere of each pore. After determining the pore sizes, the *N* pores were randomly positioned within the RVE provided that the pores were not overlapping each other. A MATLAB script was used for generating the geometry of the RVE. For such a RVE, the porosity is calculated as the total pore area divided by the RVE area.

Since the 3D printed PLA material is considered as a periodic array of the RVEs, periodic boundary conditions were applied to the RVE model. This implies that each RVE is a continuous body and two continuities must be satisfied: (a) The shape at the two opposite boundaries must remain the same, such that no separation or overlap occurs between the neighboring RVEs under deformation; and (b) the tractions at the two opposite boundaries must be continuous [30,31].

To evaluate the elastic properties, a uniaxial tension was applied to the x direction on the RVE for a finite element analysis using a commercial finite element analysis package (ABAQUS, Simulia, Providence, RI), where a Python script was developed to implement the periodic boundary conditions on the RVE. The Young’s modulus for the RVE matrix was selected as 3500MPa which was given in the material data sheet for the PLA filament used for the printing. However, the data sheet did not provide any information on the Poisson’s ratio of the PLA filament. Considering that Poisson’s ratio is approximately equal to 0.33, almost independent of the process parameters and porosity from the experimental data in Table 7, here we selected Poisson’s ratio as 0.33 for the matrix to investigate whether the macroscopic Poisson’s ratio would be dependent on the porosity in the RVE analysis. The pores in the RVE were modeled as very weak solid material, that is, Young’s modulus and Poisson’s ratio were selected respectively as 0.000001 MPa and 0.000001. The RVE were meshed using six-node plane stress elements CPS6M, and mesh convergence analysis was carried out by using seed size 0.015, 0.03, and 0.06 and checking the homogenized stresses and strains over the whole RVE, which were calculated respectively by

(1) $${\bar{\sigma }}_{ij}=\frac{1}{V}\int_{V}^{}\sigma_{ij}dV$$

and

(2) $${\bar{\varepsilon }}_{ij}=\frac{1}{V}\int_{V_{}}^{}\varepsilon_{ij}dV$$

where ${\bar{\sigma }}_{ij}$ and ${\bar{\varepsilon }}_{ij}$ denote respectively the homogenized stresses and strains in the macroscale, $\sigma_{ij}$ and $\varepsilon_{ij}$ denote respectively the microscopic stresses and strains in the RVE, and V is the total volume of the RVE. After obtaining the homogenized stresses and strains, the effective elastic properties in the macroscale, that is, the predicted elastic properties for the 3D printed PLA material, can be computed by simply using

(3) $$E_{pre}=\frac{{\bar{\sigma }}_{xx}}{{\bar{\varepsilon }}_{xx}}$$

and

(4) $$\nu_{pre}=-\frac{{\bar{\varepsilon }}_{yy}}{{\bar{\varepsilon }}_{xx}}$$

where ${\bar{\sigma }}_{xx}$ is the macroscopic stress component along *x* axis and ${\bar{\varepsilon }}_{xx}$ and ${\bar{\varepsilon }}_{yy}$ are respectively the macroscopic strain components along the *x* and *y* directions.

#### 3.3.1. Statistical Simulations

To simulate the statistical studies for the 3D printed PLA material as given in Section 2.4, a statistical approach was adopted to generate the RVEs as follows.

To construct an RVE, the sizes for the *N* pores were determined based on the actual pore size distributions for the specimens given in Table 6. The ranges of the *N* pore sizes were selected to be the same with the actual pore size ranges, that is, from 0.0388 to 1 mm. To simulate the actual pore size distribution, the sizes of the *N* pores were selectively distributed in the five size ranges in Table 6 in such a manner that most of the pores had a pore size below 0.2 mm, a medium number of pores fell into the size range between 0.2 and 0.6 mm, and the pore size larger than 0.8 mm was kept very rare. One example of such a pore size distribution pattern is given in Table 8, where the number of pores in the RVE was selected between 5 and 100. For a fixed value of *N*, within each size range, the sizes of the pores in the RVE were randomly selected from the large population of the actual pore sizes characterized by XCT.

Once the sizes of the pores were determined, the size of RVE can be calculated for a given porosity. To create a more general model, we selected the porosity values between 3% and 50% for the micromechanical modeling, which allows the RVE to predict the elastic properties for the specimens tested in our study as well as the other materials of larger porosity provided that the pore size distributions for the material also fall in the range of between 0.038 mm to 1 mm, which is determined by the upper and lower pore size limit in XCT characterizations. Once the size of the RVE was determined, the *N* pores were randomly distributed in the RVE as discussed. Three RVEs were generated for fixed values of *N* and the porosity to capture the variations of elastic properties.

#### 3.3.2. Number of Pores Required for Homogenizing the Elastic Properties of the 3D Printed PLA Material

To predict the elastic properties using the proposed micromechanical model, the first step is to check the minimum number of pores for homogenizing the effective Young’s modulus and Poisson’s ratio of the RVE. More specifically, the pores in the RVE should be sufficient for representing the microscopic details in the 3D printed PLA material; however, the number of pores should be as small as possible to ensure an economic computation.

**Figure 12:**
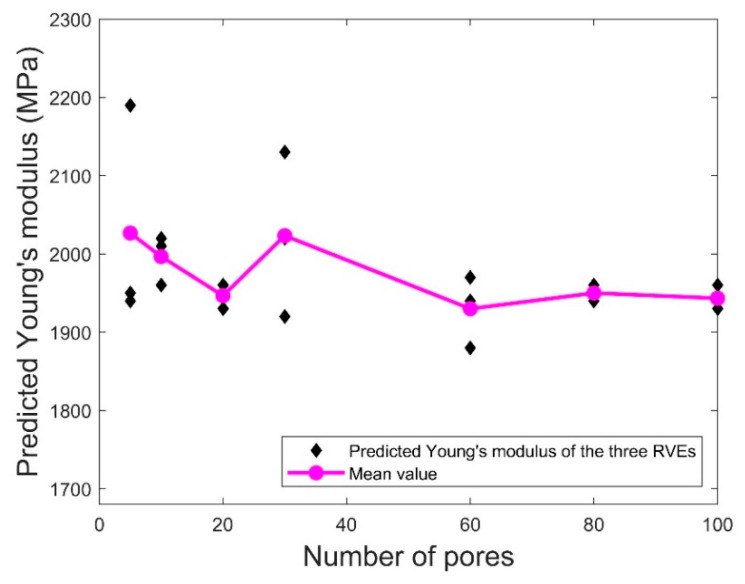
Scatter plots of the predicted Young’s modulus for porosity = 20%. Also included are the mean values of the predicted Young’s modulus of the three RVEs.

**Figure 13:**
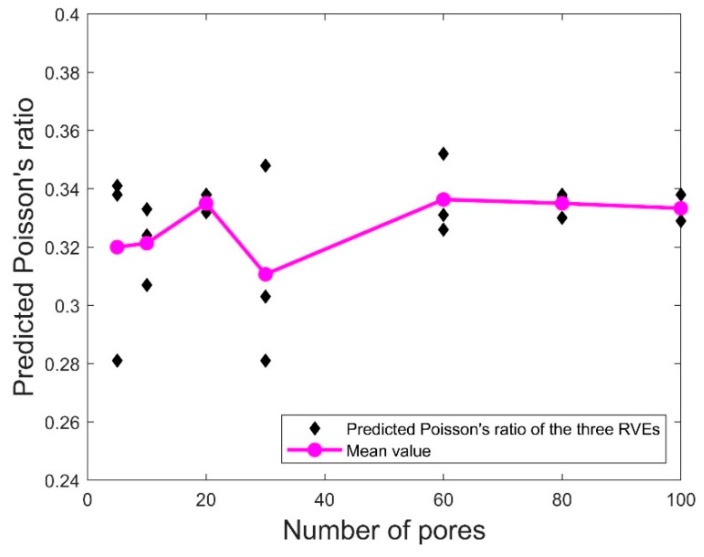
Scatter plots of the predicted Poisson’s ratio for porosity = 20%. Also included are the mean values of the predicted Poisson’s ratio of the three RVEs.

To determine the minimum number of pores in each RVE, statistical simulations were conducted using the pore size distributions following Table 8 to evaluate the elastic properties for the specimens with the selected porosity between 3% and 50% and the number of pores between 5 and 100. Figure 12 and Figure 13, respectively, show the variations of the representative predicted Young’s modulus and Poisson’s ratio of the three RVEs in set A for different numbers of pores and porosity = 20%. Also included are the mean values of the elastic properties of the three RVEs for each value of *N.* It was observed that the mean values of both elastic properties did not change very much as the number of pores reached 80. Moreover, for the number of pores below 80, the variation of the elastic properties for the three RVEs was much more serious compared to the corresponding data for *N* = 80 and 100. This indicates that 80 pores in the RVE may be sufficient for homogenizing the predicted Young’s modulus and Poisson’s ratio. For the following results, we use *N* = 80 in the RVE to predict the elastic properties.

For *N* = 80 and 100, it was also found that the mean values of the elastic properties predicted by using the pore size data for the five sets of specimens were very close to each other with a percentage difference up to 1.8%. This is not surprising due to the following reasons. As shown in Table 6, for the test specimens with different porosities, the pore size ranges are the same and the size distributions are highly similar. Considering the number of pores *N* in the RVE is a much smaller number compared to the number of pores in the real population (ranges from 464,653 to 712,525 for porosities between 4.05% and 6.32%), it is expected that adopting the pore size data from any specimen in the five sets will lead to very similar prediction results. Thus in the following sections, we select the pore size distributions of set A for predicting the elastic properties of the 3D printed PLA materials.

#### 3.3.3. Comparison with the Existing Models

In this section, the predicted elastic properties by the proposed micromechanical model are compared with two existing classical numerical works widely used for benchmarking elasticity problems involving porosity effects [33,34]. In the first model, [35] investigated a zig-zag array of circular holes with uniform sizes in an infinite matrix by assuming complex stress potentials in the form of Laurent series expansions. Specifically, the effective Young’s modulus for two specially decoupled arrays of the holes, the square array and the equilateral triangular array, are explicitly given as functions of the fraction of the holes (which can be considered as porosity) up to 50% in terms of power series fitted to the numerical results.

The second numerical model [36] applied periodic boundary conditions on an RVE to analyze the effective Young’s modulus of three patterns of circular holds, also with uniform sizes, as a function of the porosity. The three patterns included non-overlapping periodically centered holes on (a) a honeycomb lattice, (b) a triangular lattice, and(c) overlapping-allowed, randomly centered circular holes.

For our micromechanical model, the predicted elastic properties are expected to depend on the porosity as well as the pore size distributions [37]. Thus, apart from the pore size distributions described in Section 3.3.1, here we consider one more distribution of the pore sizes in the RVE for a more comprehensive study. As indicated in Table 6, over 99% of pore sizes fell into the range below 0.2 mm. To capture the large population of the smaller pores, we assumed all of the 80 pores in the RVE to have sizes between 0.0388 and 0.2 mm. The prediction results were compared with the RVE analysis using the full range of the pore sizes (between 0.0388 and 1 mm) selected as in Table 8 as well as the two existing numerical works. As before, three RVEs were generated for each type of pore size distribution and each porosity for *N* = 80.

**Figure 14:**
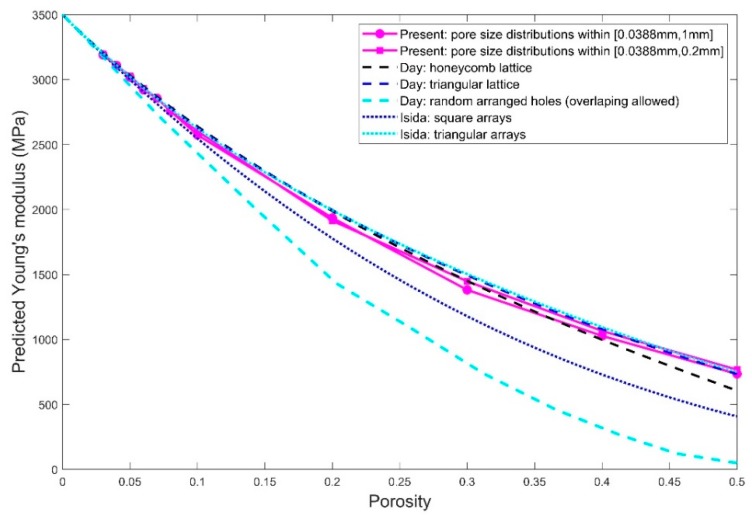
Predicted Young’s modulus against the porosity for the RVEs using both pore size distributions within [0.388 mm, 0.2 mm] and [0.388 mm, 1 mm]. Also included are the corresponding works from [35] and [36] abbreviated respectively as Isida and Day in the figure.

**Figure 15:**
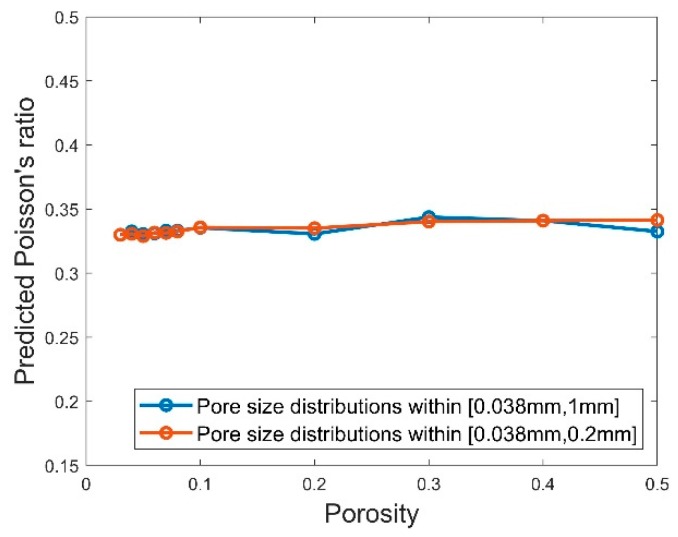
Predicted Young’s modulus against the porosity for the RVEs using pore size distributions within [0.388 mm, 0.2 mm] and [0.388 mm, 1 mm].

For the porosity between 3% and 50%, the prediction results of Young’s modulus and Poisson’s ratio are respectively given in Figure 14 and Figure 15 for the cases using the pore sizes in the full range (that is, from 0.038 to 1 mm) as well as only small pore sizes below 0.2 mm. The presented data were obtained by averaging the elastic properties calculated for the three RVEs with the randomly positioned pores. Also included are the Young’s modulus predicted by the existing models [35,36]. It is observed that the elastic properties predicted using the two pore size distributions do not differ too much, having a percentage difference up to 6.5% for larger porosity between 10% and 50% and a percentage difference up to 1.2% for smaller porosity below 10%. The prediction results using the pore sizes between 0.0388 and 0.2 mm are slightly larger compared to the ones using the full range of pore sizes. It appears that the larger pores with sizes ranging from 0.2 to 1 mm do not affect the prediction results very much, since the main contribution comes from the large population of the smaller pores (below 0.2 mm).

When it cames to the comparison with the existing models in [35,36], our predictions were generally close to the effective Young’s modulus estimated by the cases of the centered holes in honeycomb and triangular lattices in [36] as well as the case of periodic holes in triangular arrays in [35], especially for porosity lower than 40%. The predicted Young’s modulus for both pore size distributions falls between the triangular and square array of holds proposed by [35]. Compared to the pore size distributions involving the large pores (the full range of pore sizes), the RVEs using only smaller pores below 0.2 mm gave rise to predicted Young’s modulus closer to the case with the holes centered in a triangular lattice in [36] and the holes in triangular arrays in [35]. This may be as expected since [35] and [36] assume the holes to have uniform diameters and our pore size selected within 0.0388 to 0.2 mm gave rise to more uniform-like sizes compared to the other case with the pore sizes selected up to 1mm. Moreover, it is not surprising from Figure 14 that the cases with the randomly arranged holes from [36] provide Young’s modulus that is much deviated from our prediction results if we take into account that overlapped holes are allowed in [36] but strictly not allowed in our micromechanical model.

For Poisson’s ratio, it is observed from Figure 15 that the predicted values for the different values of porosity and both pore size distributions are all approximately equal to 0.33 which is the Poisson’s ratio which we used as the material property of the matrix in the micromechanical simulations. This also matches one of the conclusions in [36], which pointed out that the effective Poisson’s ratio should be approximately equal to 1/3 if the Poisson’s ratio for the matrix is taken as 1/3.

#### 3.3.4. Comparison with the Experimental Results

**Figure 16:**
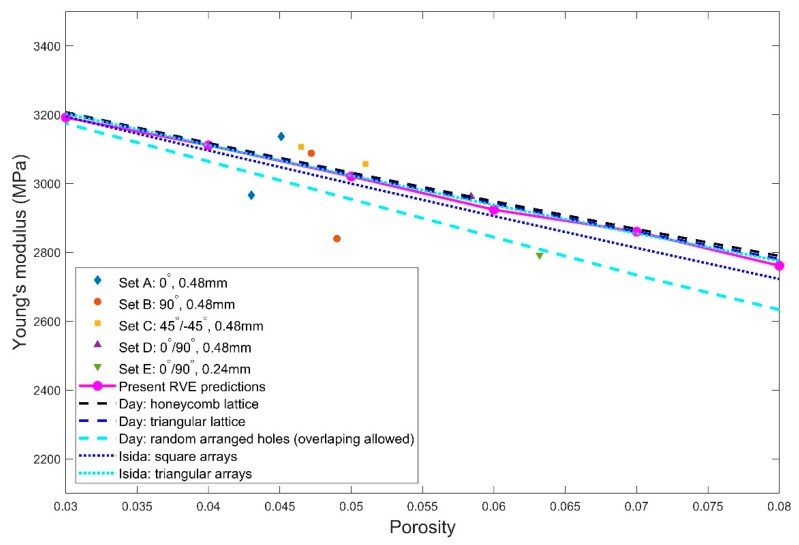
Comparison of the predicted Young’s modulus with the experimental data against porosity for the five sets of specimens (with various combinations of raster orientations and extrusion widths). The results from the existing numerical models in [35,36] are also included for reference.

Young’s modulus predicted by the proposed micromechanical model is compared with the experimental data from the tensile test in this subsection. The full range of the pore sizes between 0.0388 and 1 mm were used for the pores in the RVE to calculate the predicted Young’s modulus. The porosity for the RVE was selected between 3% and 8% to fully cover the range of the porosity characterized by XCT. For each porosity, three RVEs were generated by randomly positioning the 80 pores within the RVE and the predicted Young’s modulus for the three RVEs were averaged to be compared with the experimental Young’s modulus. Figure 16 shows the plots of both experimental and numerically predicted Young’s modulus against the porosity. The numerical data from [35,36] are also provided here for reference. It is observed that the predicted Young’s modulus agreed well with the experimental data, with the percentage difference between the corresponding data points less than 7.9%. For such low porosities, Young’s modulus predicted by the micromechanical model also agreed very well with all of the five cases in [35,36]. The comparison in Figure 16 demonstrates that the proposed micromechanical model can be used to predict the effective elastic properties of the 3D printed PLA material based on a known porosity.

## 4. Summary and Conclusions

The present study adopted experiments and micromechanical modeling to correlate microscopic details of the internal pores and mechanical properties of 3D printed polymeric material. Firstly, tensile test specimens, with various raster orientations and raster widths, were fabricated by a desktop 3D printer using the fused deposition modeling technique. Subsequently, the three-dimensional details of the internal pores for the 3D printed specimens, including size, shape, and spatial location of the pores as well as porosity, were quantitatively characterized using X-ray computed tomography (XCT). The tensile tests were then performed to characterize the mechanical properties of the specimens. Last but not least, micromechanical modeling was conducted to develop a representative volume element (RVE) for predicting the macroscopic mechanical properties based on the microscopic details of the internal pores characterized by XCT.

As expected, the results for XCT and mechanical characterization showed that the specimens of lower porosity have better mechanical properties. The specimens in all of the selected process parameters demonstrated very similar pore size distributions, that is, over 99% internal pores of the 3D printed material had small sizes below 0.2 mm and less than 1% of pore sizes fell into the range of 0.2 to 1 mm. The actual pore size distributions were used to generate the RVEs and in turn to predict the macroscopic elastic properties. The prediction results for the elastic properties showed good agreement with the corresponding experimental data where the percentage difference was not larger than 7.9%. The predicted elastic properties also agreed well with two existing numerical works. The proposed micromechanical model has demonstrated itself as a potential tool for predicting elastic properties for the future designer. This provides a possibility of saving the material from undergoing destructive testing.

The present micromechanical modeling was conducted based on two dimensional assumptions that the pores in the RVEs have circular shapes whose diameters were taken by measuring the circumscribed sphere of the selected pores in XCT characterizations. It could be improved in future works by taking into account the three dimensional details of pore shapes, such as sphericity and the aspect ratio, as well as the spatial alignment and distribution of the pores.

## Figures and Tables

**Table 1:** Process parameter sets for fabricating the tensile specimens.

Specimen Set	Raster Orientation	Extrusion Width
A	0°	0.48 mm
B	90°	0.48 mm
C	45°/–45°	0.48 mm
D	0°/90°	0.48 mm
E	0°/90°	0.24 mm

**Table 2:** Pore size distribution analyzed using various pore size limits.

Range of Pore Size (mm)	Number of Pores		
	UPL = 1 mm	UPL = 1.8 mm	UPL = 3.6 mm
**0.0388~0.2**	706,591	706,658	685,320
**0.2~0.4**	2982	2974	2983
**0.4~0.6**	2314	2260	2223
**0.6~0.8**	244	239	236
**0.8~1**	393	399	412
**1~1.8**	N.A.	323	321
**1.8~3.6**	N.A.	N.A.	234
**Total number of pores**	712,524	712,853	691,729

**Table 3:** Values of porosity calculated using various pore size limits (UPL).

UPL (mm)	Largest Pore Size (mm)	Porosity (%)
1	0.9997	6.32
1.8	1.7961	6.53
3.6	3.5971	6.66

**Table 4:** Porosity results for specimens with various raster orientations and the extrusion width selected as 0.48 mm.

Specimen Set	Raster Orientation	Specimen Number	Porosity (%)
A	0°	#1	4.51
		#2	4.05
B	90°	#1	4.72
		#2	4.90
C	45°/−45°	#1	4.65
		#2	5.10

**Table 5:** Porosity results for specimens with various extrusion widths and the raster orientation selected as 0°/90°.

Specimen Set	Extrusion Width (mm)	Porosity (%)
D	0.48	5.84
E	0.24	6.32

**Table 6:** Pore size distribution for the representative specimen in each set of process parameters.

Range of Pore Size (mm)	Number of Pores				
	Set A	Set B	Set C	Set D	Set E
**0.0388~0.2**	460,872	553,553	479,193	689,548	706,591
**0.2~0.4**	3248	446	552	1222	2982
**0.4~0.6**	466	61	83	236	2314
**0.6~0.8**	43	37	32	97	244
**0.8~1**	23	45	32	183	393
Total number of pores	464,652	554,142	479,892	691,286	712,524

**Table 7:** Mechanical properties for the 3D printed PLA material.

Specimen Set	Young’s Modulus (MPa)	Poisson’s Ratio	Ultimate Strength (MPa)
A	3170 ± 78	0.331 ± 0.002	60.3 ± 2.1
B	2970 ± 102	0.333 ± 0.002	55.9 ± 0.7
C	3086 ± 34	0.328 ± 0.002	59.2 ± 1.8
D	2809 ± 130	0.331 ± 0.001	56.1 ± 3.8
E	2697 ± 78	0.327 ± 0.001	54.4 ± 1.5

**Table 8:** Pore size distribution in the RVE for *N* between 5 and 100.

Number of Pores N	Number of Pores in Each Range of Pore Size (in mm)				
	0.0388~0.2	0.2~0.4	0.4~0.6	0.6~0.8	0.8~1
5	3	0	1	0	1
10	6	2	1	0	1
20	12	4	2	1	1
30	22	4	2	1	1
60	36	12	6	3	3
80	48	16	8	4	4
100	60	20	10	5	5
